# A Head View-Invariant Representation of Gaze Direction in Anterior Superior Temporal Sulcus

**DOI:** 10.1016/j.cub.2011.09.025

**Published:** 2011-11-08

**Authors:** Johan D. Carlin, Andrew J. Calder, Nikolaus Kriegeskorte, Hamed Nili, James B. Rowe

**Affiliations:** 1 Medical Research Council Cognition and Brain Sciences Unit, 15 Chaucer Road, Cambridge CB2 7EF, UK; 2 Department of Clinical Neurosciences, University of Cambridge, Addenbrooke's Hospital, Cambridge CB2 2QQ, UK; 3 Behavioural and Clinical Neuroscience Institute, University of Cambridge, Cambridge CB2 3EB, UK

## Abstract

Humans show a remarkable ability to discriminate others' gaze direction, even though a given direction can be conveyed by many physically dissimilar configurations of different eye positions and head views. For example, eye contact can be signaled by a rightward glance in a left-turned head or by direct gaze in a front-facing head. Such acute gaze discrimination implies considerable perceptual invariance. Previous human research found that superior temporal sulcus (STS) responds preferentially to gaze shifts [1], but the underlying representation that supports such general responsiveness remains poorly understood. Using multivariate pattern analysis (MVPA) of human functional magnetic resonance imaging (fMRI) data, we tested whether STS contains a higher-order, head view-invariant code for gaze direction. The results revealed a finely graded gaze direction code in right anterior STS that was invariant to head view and physical image features. Further analyses revealed similar gaze effects in left anterior STS and precuneus. Our results suggest that anterior STS codes the direction of another's attention regardless of how this information is conveyed and demonstrate how high-level face areas carry out fine-grained, perceptually relevant discrimination through invariance to other face features.

## Highlights

► Response patterns in superior temporal sulcus (STS) code perceived gaze direction ► Gaze codes are invariant to head view and physical image features in anterior STS ► However, such socially irrelevant features do influence gaze codes in posterior STS ► Anterior STS represents where others attend, regardless of how this is conveyed

## Results and Discussion

**Figure 1:**
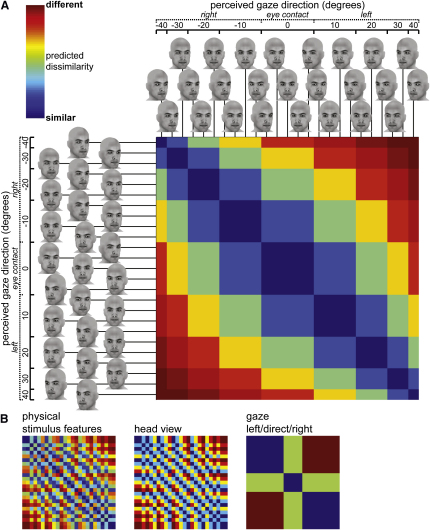
Stimuli and Predicted Dissimilarity Matrices (A) Predicted view-invariant gaze direction dissimilarity structure across the 25 computer-generated faces. The faces are sorted according to the nine distinct gaze directions in the stimulus set (left 40° to right 40° rotation), which were created by incrementally varying head view and eye position relative to the head (five increments between left 20° and right 20° for both). (B) Predicted dissimilarity structures for the same faces based on alternative accounts of the data corresponding to their physical stimulus features (1-r across image grayscale intensities), head view, and qualitative gaze direction (left/direct/right gaze) ignoring quantitative differences between angles of left and right gaze. Dissimilarity matrices are sorted as in (A).

We designed a set of 25 computer-generated faces where nine gaze directions were conveyed by multiple, physically dissimilar configurations of different head views and eye positions (Figure 1A). This allowed us to disentangle functional magnetic resonance imaging (fMRI) responses consistent with head view-invariant representations of gaze direction from responses related to the faces' other physical features [2]. Previous reports of superior temporal sulcus (STS) involvement in perception of gaze and head view used faces in which eye position or head view were manipulated in isolation [3–5]. Such designs cannot address the issue of view-invariant coding of gaze because the degree of eye position or head view change defines the degree of gaze direction change. Moreover, previous attempts to identify view-invariant gaze codes using conventional univariate analysis of smoothed fMRI data have produced inconsistent results and did not observe gaze effects in STS [6, 7]. This is perhaps unsurprising, because macaque STS neurons that are selective for head view and gaze direction are organized into small patches [8, 9] beyond the likely resolution of conventional fMRI analysis methods. Recently, multivariate pattern analysis (MVPA) has been used to identify other visual representations thought to be coded at similarly small spatial scales, including direction-specific motion responses in early visual cortex [10, 11]. Here, we applied novel MVPA methods (representational similarity analysis [12]) to high-resolution fMRI data in order to reveal response pattern codes for view-invariant gaze direction.

### Representational Similarity Analysis of Gaze Codes

**Figure 2:**
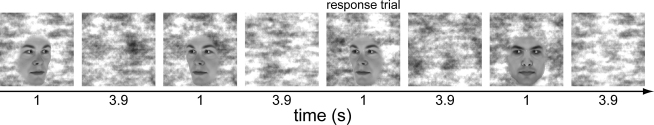
An Example Trial Sequence from the fMRI Experiment The faces were presented in random order in a rapid event-related design. Participants maintained fixation on a central cross. The faces were presented so that the cross fell on the bridge of the nose of each face to minimize eye movements during the task. The 25 head/eye position configurations were posed by two identities (50 images total). Each was presented three times in five independently randomized sets (150 experimental trials presented over 11 min per set; 750 trials in total over 55 min). Each trial comprised a face (1 s) followed by an intertrial interval (2.9 s). Fifteen randomly selected trials in each set were immediately followed by a second presentation of the same face (75 added trials in total). Participants were asked to identify repetitions with a button response before the onset of the next trial (one-back task). Response trials were equally sampled from all head/eye position configurations and were modeled with a separate regressor of no interest in the first-level fMRI model. At the end of each set, participants viewed a feedback screen (20 s) that summarized their hit rates and false alarm rates for that set. See Supplemental Experimental Procedures for a complete account of stimulus design and procedure.

Eighteen human participants carried out a one-back matching task while viewing the gazing faces in a rapid event-related fMRI experiment (for details, see Figure 1, Figure 2, and Experimental Procedures). Eye tracking data were also acquired to rule out confounding influences of eye movements (see Supplemental Experimental Procedures available online).

We extracted each participant's responses to each face (t contrast maps against baseline) to estimate response pattern dissimilarities between each face pair (1-Pearson r across voxels). These dissimilarities were compared to a predicted dissimilarity structure for view-invariant gaze direction and to other dissimilarity structures representing alternative accounts of the data (Figure 1B). We quantified the relationship between the response pattern dissimilarities and the predicted dissimilarities as the Spearman rank correlation across all face pairs. This representational similarity analysis [12] was carried out in single participants using a searchlight algorithm [13] (5 mm radius sphere) that localizes response pattern effects to local voxel neighborhoods.

Individual participants' results for each response-predictor comparison were spatially normalized to a common template, smoothed, and tested for group effects using a permutation test (Experimental Procedures). Based on previous evidence for right-lateralized gaze responses in human STS [3, 4, 14], we report all p values in the primary analysis corrected for multiple comparisons within the anatomically defined right STS region (p < 0.05, familywise error [FWE]; Figure S1A, 4598 voxels). For completeness, we also carried out exploratory analyses of left STS and the full gray-matter-masked volume.

### Right STS Gaze Codes Are Invariant to Head View and Physical Stimulus Features

**Figure 3:**
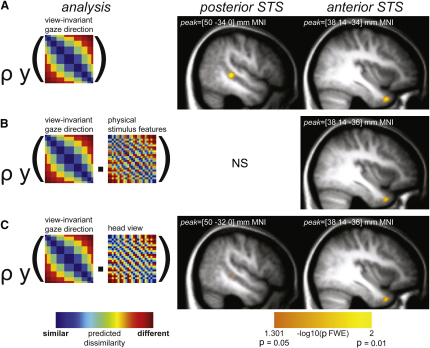
Regions with Pattern Responses to the Gazing Faces Spearman correlations of partial correlation effects across participants (n = 18, p < 0.05, familywise error [FWE] corrected for right STS; Figure S1A) are shown overlaid on the sample's mean structural volume. (A) Response pattern dissimilarities in anterior and posterior STS are explained by the view-invariant gaze direction predictor. (B) Gaze direction responses in anterior STS alone are found for the same predictor when controlling for physical stimulus features. (C) Similarly, gaze direction responses in anterior STS for the view-invariant gaze predictor are unaffected when controlling for head view, whereas responses in posterior STS are reduced.

Response patterns in right anterior (p = 0.013) and posterior (p = 0.006) STS showed a consistent relationship with the view-invariant gaze direction predictor (Figure 3A). Complementary functional region of interest analyses of right STS revealed moderate independently estimated effect sizes in these regions (r = 0.39 for anterior STS, r = 0.42 for posterior; Figure S1B). Although these effects suggest that both regions code the direction of another's gaze, it was important to correct for unavoidable correlations between the view-invariant gaze direction predictor and alternative predictors derived from the faces' physical stimulus features (1-r across image grayscale intensities) or head view (correlation between gaze direction and physical stimulus features r = 0.37, correlation between gaze direction and head view r = 0.36; Figure 1B). Note that the relationship with both is because the faces' physical stimulus features were almost entirely explained by head view (r = 0.99).

To exclude the contribution of these additional facial properties, we computed a further correlation between the view-invariant gaze direction predictor and the response pattern dissimilarities, this time partialing out any correlation between physical stimulus features and the response pattern dissimilarities (partial Spearman correlation). Only the perceived gaze direction effect in anterior STS remained significant when the influence of physical stimulus features was removed (p = 0.018; Figure 3B). Similarly, removing the influence of head view did not disrupt the effect of the view-invariant gaze direction predictor in anterior STS (p = 0.016) but produced only a weakly significant effect in posterior STS (p = 0.045; Figure 3C). Indeed, posterior STS showed a significant relationship with the predictor derived from the faces' physical stimulus features (p = 0.048) and a near-significant relationship with the predictor derived from head view (p = 0.08). Thus, gaze direction responses in posterior STS were influenced by physical stimulus features, which corresponded largely to variation in head view, whereas gaze direction responses in anterior STS were invariant to these facial properties.

### Right STS Gaze Codes Are Fine Grained

If gaze codes in STS play a role in supporting perceptual performance, such codes should mirror human sensitivity to fine-grained gaze direction distinctions [15]. We tested this by comparing the original view-invariant gaze predictor representing nine gaze directions to a left/direct/right gaze predictor that distinguished between three qualitative gaze directions, while ignoring continuous information about the degree to which gaze is averted left or right (Figure 1B). Partial correlation analysis showed that the effects of the original view-invariant gaze predictor remained after removing the influence of the left/direct/right gaze predictor (anterior STS p = 0.016, posterior STS p = 0.018; Figure S1C). Thus, the reported view-invariant gaze direction effects cannot be explained by simpler gaze representations. Instead, gaze direction codes in STS contained fine-grained information about both the direction and the degree to which gaze is averted.

### Gaze Codes in Left STS and Precuneus

An exploratory analysis of left STS revealed similar evidence of view-invariant coding of gaze direction in left anterior STS (Table S1). There were no significant effects in left posterior STS (p > 0.19). View-invariant representations of gaze direction in anterior STS may therefore be bilateral.

A further analysis of the full gray-matter-masked volume also revealed view-invariant gaze direction codes in precuneus, which survived all control analyses reported above (Table S1). Precuneus and STS are monosynaptically connected in macaques [16], and precuneus has previously been implicated in head/gaze following [17] and in attentional orienting [18], which suggests that gaze codes here may reflect gaze-cued shifts in attention [19]. Eye tracking analyses suggested that participants were fixating well (Supplemental Experimental Procedures), so these precuneus effects are likely driven by covert attentional shifts rather than overt eye movements.

### Participant-Specific Gaze Codes

Our experimental design assumes that perceived gaze direction can be approximated by the sum angle of head view and eye position relative to the head (Figure 1) [2]. However, human gaze discrimination performance can be subtly biased by head view [20, 21]. We therefore carried out a follow-up behavioral experiment to assess whether the standard view-invariant gaze predictor we used was a good match for the participants' individual gaze discrimination performance. Each participant in the fMRI experiment carried out a subsequent task where they indicated the perceived gaze direction of the faces they had viewed in the scanner. Difference scores between the perceived gaze direction for the different face pairs were then compared to the standard view-invariant gaze predictor (Supplemental Experimental Procedures). Gaze discrimination performance was well explained by the generic view-invariant gaze direction predictor (median Spearman r = 0.90, 95% confidence = 0.87–0.93, bootstrap test), and this relationship survived removing the influence of each of the alternative predictors discussed above (Figure S1E).

We also repeated the fMRI analyses using the participant-specific gaze discrimination predictors in place of the standard view-invariant gaze direction predictor, and obtained comparable results (Table S1). Thus, participants' gaze discrimination performance was well approximated by the standard view-invariant gaze predictor, and the neural responses to the gazing faces were similarly explained by the standard and participant-specific gaze predictors.

### Conclusions

This study provides the first evidence that human anterior STS contains a fine-grained, view-invariant code of perceived gaze direction. We also observed similar gaze effects in precuneus, which may reflect attentional orienting responses to gaze [19]. Our results do not rule out the existence of view-specific codes for particular head-gaze configurations but rather demonstrate that gaze perception is not achieved using such view-specific representations alone. Our results are consistent with the hypothesis that gaze perception is achieved through a high-level, view-invariant code of the direction of another's social attention in anterior STS.

The representational content of right posterior STS is distinct from anterior STS. Although the view-invariant gaze predictor also identified this region, this was largely accounted for by the modest correlation between this predictor and the faces' physical facial properties or head view, which showed significant or borderline relationships with the right posterior STS. This is consistent with recent work showing that response patterns in posterior STS can be used to distinguish head view [5]. The preferential involvement of anterior STS in view-invariant representations of gaze direction was further underlined by the analysis of left STS, which identified the anterior region alone. Our results are thus consistent with previous reports that right posterior STS is responsive to different gaze directions and head views [1, 5], but view-invariant gaze direction codes appear most prevalent in anterior STS.

Collectively, our results suggest a hierarchical processing stream for gaze perception, with increasing invariance to gaze-irrelevant features from posterior to anterior STS. Such a processing hierarchy would be consistent with recent evidence from neurons responsive to face identity in the macaque temporal lobe [22], where invariance to head view increases from middle STS to anterior inferotemporal cortex. Similarly, neurons tuned to specific head views in anterior STS also frequently respond to gaze direction [23–25], whereas neurons with head view tunings in middle STS generally do not [25]. Such hierarchical progressions toward view invariance may therefore be a general property of high-level face representations, regardless of whether these hierarchies serve to extract face identity or the direction of another's gaze.

In conclusion, response patterns in human anterior STS are not coded according to any readily observable visual face features but rather according to the direction of another person's gaze, irrespective of head view.

## Experimental Procedures

### Participants

Twenty-three right-handed participants with normal or corrected-to-normal vision were recruited for the study. Participants provided informed consent as part of a protocol approved by the Cambridge Psychology Research Ethics Committee. Five participants were removed from further analysis: two failed to complete the experiment, two fell asleep and displayed excessive head motion, and one failed to maintain fixation (Supplemental Experimental Procedures). This left 18 participants (five male, mean age 24, age range 18–36).

### Imaging Acquisition

Scanning was carried out at the MRC Cognition and Brain Sciences Unit (Cambridge) using a 3 T TIM Trio Magnetic Resonance Imaging scanner (Siemens), with a head coil gradient set. Functional data were collected using high-resolution echo planar T2^∗^-weighted imaging (40 oblique axial slices, repetition time [TR] 2490 ms, echo time [TE] 30 ms, in-plane resolution 2 × 2 mm, slice thickness 2 mm plus a 25% slice gap, 192 × 192 mm field of view). The acquisition window was tilted up approximately 30° from the horizontal plane to provide complete coverage of the occipital and temporal lobes. All volumes were collected in a single, continuous run for each participant. The initial six volumes from the run were discarded to allow for T1 equilibration effects. T1-weighted structural images were also acquired (MPRAGE, 1 mm isotropic voxels).

### Imaging Analysis

Preprocessing of the fMRI data was carried out using Statistical Parametric Mapping 5 (SPM5; http://www.fil.ion.ucl.ac.uk/spm/). Structural volumes were segmented into gray- and white-matter partitions and normalized to the Montreal Neurological Institute (MNI) template using combined segmentation and normalization routines. All functional volumes were realigned to the first nondiscarded volume, slice time corrected, and coregistered to the T1 structural volume. The functional volumes remained unsmoothed and in their native space for participant-specific generalized linear modeling. Each set was modeled with a separate set of regressors for each head/eye configuration (25, collapsing across the two face identities), false alarms, and repeat trials. We also included scan nulling regressors to eliminate the effects of excessively noisy volumes [26, 27]. The experimental predictors were convolved with a canonical hemodynamic response function, and contrast images for each individual condition against the implicit baseline were generated based on the fitted responses. The resulting T contrast volumes were gray-matter-masked using the tissue probability maps generated by the segmentation processing stage and were used as inputs for representational similarity analysis.

Representational similarity analyses were carried out using custom code developed using Python and PyMVPA [28]. The voxels within each searchlight and each set were correlated across conditions (1-Pearson r), and the resulting 1-correlation matrix was averaged across the five sets to produce a final response pattern dissimilarity matrix for that searchlight. The data dissimilarities were then compared to a set of hypothesis-based predictors using the Spearman rank correlation or partial Spearman rank correlation. In all cases, the resulting correlation coefficient was Fisher transformed and mapped back to the central voxel in the searchlight, yielding a descriptive individual subject map that was entered into a group analysis. This two-stage summary statistics procedure resembles that used in conventional univariate fMRI group analysis [29]. The individual subject maps were normalized to the MNI template and were smoothed to overcome errors in intersubject alignment (10 mm full width at half mean [FWHM]). The resulting volumes were entered into a permutation-based random-effects analysis using statistical nonparametric mapping [30] (SnPM; 10,000 permutations, 10 mm FWHM variance smoothing). The use of nonparametric tests avoids distributional assumptions regarding the nature of the descriptive maps and avoids inherent problems in applying standard SPM5 FWE correction based on random Gaussian fields to discontinuous gray-matter-masked data.

## Supplemental Information

Document S1. One Figure, One Table, and Supplemental Experimental Procedures
