# Supplementary material for: A Head View-Invariant Representation of Gaze Direction in Anterior Superior Temporal Sulcus
Source: Curr Biol. 2011 Nov 8;21(21-2):1817–21. doi: 10.1016/j.cub.2011.09.025 (PMC3267037; doi:10.1016/j.cub.2011.09.025)
Supplement: Document S1. One Figure, One Table, and Supplemental Experimental Procedures [file mmc1.pdf]

Current Biology, Volume *21*

Supplemental Information

A Head View-Invariant Representation of Gaze Direction in Anterior Superior Temporal Sulcus

Johan D. Carlin, Andrew J. Calder, Nikolaus Kriegeskorte, Hamed Nili, and James B. Rowe

Supplemental Inventory

Figure S1. Supplemental results relating to Figure 3.

Table S1. Analyses of additional regions and tests of the performance of a participant-specific gaze discrimination predictor in place of the standard view-invariant gaze direction predictor (related to Figure 3).

Supplemental Experimental Procedures

Stimulus Design and Presentation. Description of the methods employed to generate the face images used in the experiment, and the exact parameters under which they were presented during the experiment (related to Figure 1).

Behavioral Performance. Description of control analysis of in-scanner behavioral task (related to Figure 1).

Eye Tracking. Description of acquisition and control analysis of in-scanner eye tracking data (related to Figure 1).

Gaze Discrimination Experiment. Description of control experiment to support our conception of gaze direction as the sum of head view and eye view relative to head view (related to Figure 2).

Supplemental References

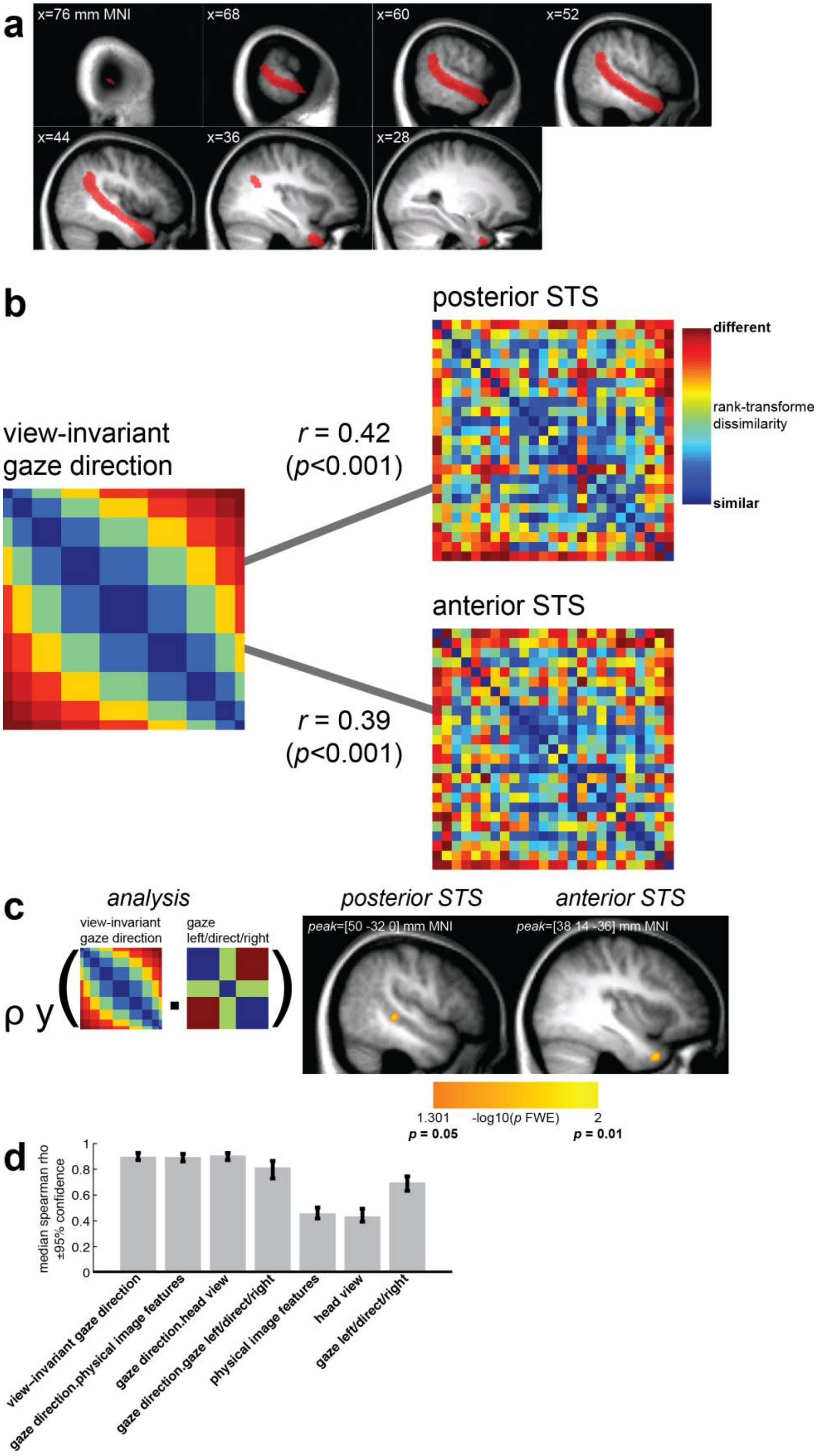
a
x=76 mm MNI
x=68
x=60
x=52
x=44
x=36
x=28
b
posterior STS
different
rank-transforme
dissimilarity
similar
view-invariant
gaze direction
r = 0.42
(p<0.001)
anterior STS
r = 0.39
(p<0.001)
c
analysis
view-invariant
gaze direction
gaze
left/direct/right
ρ y
posterior STS
anterior STS
peak=[50 -32 0] mm MNI
peak=[38 14 -36] mm MNI
1.301
-log10(p FWE)
2
p = 0.05
p = 0.01
d
median spearman rho
±95% confidence
1
0.8
0.6
0.4
0.2
0
view–invariant gaze direction
gaze direction.physical image features
gaze direction.head view
gaze direction.gaze left/direct/right
physical image features
head view
gaze left/direct/right

Figure S1.

Figure S1. Supplemental Results Relating to Figure 3

(A) Spatial extent of the right STS anatomical mask, shown overlaid on the sample's mean T1 volume.

(B) Independently estimated correlation between the view-invariant gaze direction predictor and response pattern dissimilarities in anterior and posterior right STS regions. Regions of interest (ROI) were defined using a leave-one-set-out procedure. We carried out a group analysis (similar parameters as main analysis) separately for the ROI-defining data in each unique split (4 of 5 sets) of the data to identify response pattern dissimilarities that were explained by view-invariant gaze direction. Responses to each set were estimated in five separate first-level models with 7 discarded volumes (17.43 s) separating each model to ensure independent estimates. Statistical thresholds for ROI definition varied between splits ($p < 0.01$ to $p < 0.05$, uncorrected). The only regions that appeared consistently across splits were anterior STS (mean [33.2, 10.0, -41.2] mm MNI, standard deviation [1.0, 5.1, 1.0]), and posterior STS (mean [46.4, -29.8, 4.0] mm MNI, standard deviation [3.2, 3.5, 4.2]). To better accommodate alignment errors across participants, we identified the participant-specific peak within a 10 mm radius of each group peak using ROI-defining data only. Subsequent tests of the identified ROIs were carried out separately for each split (e.g., ROIs defined using sets 1-4 were tested using set 5). We generated the illustrated response pattern dissimilarities for anterior and posterior STS by first averaging each participant's dissimilarities for each ROI across the 5 independent test splits, and then averaging the resulting ROI dissimilarity matrices across participants. It can be seen that both anterior and posterior STS showed consistent effects of view-invariant gaze direction in the independent test data (*p* values were defined using a permutation test where the order of the matrices were shuffled without replacement 10000 times [1]).

(C) Fine-grained gaze direction codes in right STS. Regions with consistent pattern responses (partial Spearman correlation) across participants ($n = 18$, $p < 0.05$ FWE). View-invariant gaze direction responses in anterior and posterior right STS remain when the influence of a qualitative distinction between gaze left/direct/right is removed.

(D) Gaze direction discrimination. Median Spearman correlations (bars 1,5-7) and median partial Spearman correlations (bars 2-4) across the participants (+/- 95% bootstrap confidence intervals). The participants' gaze discrimination performance was most strongly correlated with the view-invariant gaze direction predictor. Although performance was also moderately correlated with physical image features and head view, the strength of the relationship between discrimination performance and the view-invariant gaze direction predictor is relatively unaffected by partialling out the influence of these alternative predictors.

Table S1. Analyses of Additional Regions and Tests of the Performance of a Participant-Specific Gaze Discrimination Predictor in Place of the Standard View-Invariant Gaze Direction Predictor (Related to Figure 3)

Analysis	Comparison	Region	*p* (FWE)	Peak (mm MNI)		
				x	*y*	*z*
right STS, 4598 voxels (p<0.05)	View-invariant gaze direction	posterior STS	0.01	50	-34	0
		anterior STS	0.01	38	14	-34
	… partialling out physical stimulus features	anterior STS	0.02	38	14	-36
	… partialling out head view	posterior STS	0.05	50	-32	0
		anterior STS	0.02	38	14	-36
	… partialling out gaze left/direct/right	posterior STS	0.02	50	-32	0
		anterior STS	0.02	38	14	-36
	Physical stimulus features	posterior STS	0.05	50	-32	2
	Participant-specific gaze discrimination	posterior STS	0.01	50	-34	0
		anterior STS	0.04	38	12	-36
	… partialling out physical stimulus features	anterior STS	0.05	38	12	-36
		posterior STS	0.06	50	-34	0
	… partialling out head view	posterior STS	0.04	50	-34	0
		anterior STS	0.05	38	12	-36
	… partialling out gaze left/direct/right	posterior STS	0.01	50	-34	0
		anterior STS	0.04	38	12	-36
left STS, 4442 voxels (p<0.1 to illustrate marginally significant effects mirroring those observed in right STS)	View-invariant gaze direction	anterior STS	0.04	-60	-8	-16
	… partialling out physical stimulus features	anterior STS	0.05	-62	-6	-16
	… partialling out head view	anterior STS	0.05	-62	-6	-16
	… partialling out gaze left/direct/right	anterior STS	0.03	-60	-8	-16
		middle STS	0.07	-66	-26	-8
	Participant-specific gaze discrimination	anterior STS	0.05	-60	-6	-16
	… partialling out physical stimulus features	anterior STS	0.07	-62	-6	-14
	… partialling out head view	anterior STS	0.05	-62	-6	-14
	… partialling out gaze left/direct/right	anterior STS	0.03	-60	-8	-16
		middle STS	0.06	-66	-26	-8
whole brain analysis, 134174 voxels (p<0.05)	View-invariant gaze direction	precuneus	0.01	4	-58	30
		cerebellum	0.04	-16	-76	-50
		cingulate gyrus	0.05	2	-22	32
	… partialling out physical stimulus features	precuneus	0.02	4	-58	30
	… partialling out head view	precuneus	0.01	4	-58	30
		cerebellum	0.05	-18	-76	-50
	… partialling out gaze left/direct/right	precuneus	0.03	4	-58	30
		cerebellum	0.02	-16	-76	-50
		occipital pole	0.04	-10	-98	2
	Participant-specific gaze discrimination	precuneus	0.01	4	-58	30
		posterior STS	0.04	50	-34	0
	… partialling out physical stimulus features	precuneus	0.03	4	-58	30
	… partialling out head view	precuneus	0.02	4	-58	30
		cerebellum	0.04	-18	-76	-50
	… partialling out gaze left/direct/right	precuneus	0.01	6	-58	30
		posterior STS	0.03	50	-34	0
		anterior STS	0.04	-60	-12	-26

Peak MNI coordinates are shown with p values FWE-corrected for regions as indicated by the analysis column.

Supplemental Experimental Procedures

Stimulus Design and Presentation

We used Poser 6 (Curious Labs Inc. Santa Cruz, CA) to create grey scale face images of two identities, each displaying 25 head-gaze configurations. Each face varied in horizontal head view (5 increments from left 20° to right 20°), horizontal eye position relative to the head (same increments as for head view), and identity (2 faces). The faces were processed in Matlab (The MathWorks, Inc., Natick, MA) to achieve similar luminance histograms, and were cropped to ensure that each face appeared in a similar retinal area. Cropping was achieved with a smooth border, and the resulting face was superimposed on a background texture that varied across conditions and across repetitions of the same face (Figure 1a). The background textures were created by Fourier-scrambling each of the 50 faces separately. The inclusion of the background texture served to reduce the influence of low-level physical differences between the conditions, and to increase the difficulty level of the one-back behavioral task. Stimuli were back-projected onto a screen in the scanner which participants viewed via a tilted mirror. The stimuli extended a 6° visual angle including the background texture, and approximately 3° horizontally by 4° vertically without it. The experiment was controlled using Matlab and the Psychophysics toolbox [2].

Behavioral Performance

Participants carried out a one-back face matching task whilst in the scanner. The task instruction was to respond to any repetition of the same face (same identity and head view/eye position configuration) while ignoring the scrambled backgrounds, which did not repeat. Accuracy was relatively high across the sample (mean 77%, standard error 3%), with low false alarm rates (mean 4.5% of trials, standard error 2.3%), and high sensitivity (mean d' 2.52, standard error 0.13). The large number of different head view/eye position configurations (25) relative to the number of response trials (75 per participant) meant that there was insufficient behavioral data available to model each of the 25 configurations separately. Thus, we pooled the available response trials according to gaze direction, and calculated accuracy scores for each of the 9 gaze directions. Repeated-measures ANOVA revealed no significant accuracy effects of gaze direction (p>0.23), suggesting that attentional or performance differences did not confound our fMRI analysis.

Eye Tracking

All participants' eye movements were monitored in the scanner using an infrared video-based eye tracker (50 Hz acquisition, Sensomotoric Instruments, Germany). Successful calibrations were obtained for 10 participants out of the final sample of 18. The remaining participants were excluded from the eye tracking analysis. On-line visual inspection of the eye tracking monitor suggested that these participants were maintaining their gaze at the fixation cross. Eye tracking data were analysed using custom code developed in Matlab.

To measure stimulus-induced eye movements, we analysed how the horizontal and vertical fixation position shifted between the start and the end of each stimulus presentation. This fixation shift was analysed using ANOVAs for each individual participant. We used a one-way ANOVA where the faces were labelled according to perceived gaze direction. One participant showed an effect of gaze direction on horizontal fixation shifts ($F_{(711,8)}$=2.37, p = 0.016). This participant was removed from further analyses of the fMRI data. No other horizontal or vertical

fixation shift effects were significant in single subjects, or in a group analysis. Thus, our participants were able to comply with the instruction to maintain fixation on the central cross.

Gaze Discrimination Experiment

At the end of the fMRI experiment, all participants carried out a follow-up gaze direction discrimination experiment outside the scanner. Participants viewed the stimuli from the main fMRI experiment from a fixed position in a head rest. On each trial, participants were asked to indicate the perceived direction of gaze by rotating a pointer in the horizontal plane; this pointer was positioned in front of the screen directly underneath the stimulus. Separate representational dissimilarity matrices were then generated from each participant's discrimination data by computing the difference between the mean pointer positions for each pair of head view/eye position configurations. The relationship between each participant's perceptual discrimination and the predicted dissimilarity structures was then estimated using a similar procedure to the main fMRI analysis. Inference was carried out at the group level, using confidence intervals based on bootstrap testing of the median (bootci function in Matlab, 95% interval, 10000 samples).

Participants' gaze discrimination performance was highly correlated with the view-invariant gaze direction predictor (Figure S1d), and to a lesser extent also with physical image features and head view. The correlation between discrimination performance and the view-invariant gaze direction predictor was largely unaffected when the influence of each of these alternative predictors was partialled out. The standard gaze direction predictor was thus a good approximation for participants' perceived gaze direction in this stimulus set.

Supplemental References

1. Kriegeskorte, N., Mur, M., and Bandettini, P. (2008). Representational similarity analysis - connecting the branches of systems neuroscience. Front. Sys. Neurosci. *2*, 1-28.
2. Brainard, D. (1997). The psychophysics toolbox. Spat. Vis. *10*, 433-436.
